# Morphologic and mechanical adaptive variations in *Saiga tatarica* calcaneus: A model for interpreting the bone functional adaptation of wild artiodactyl in captivity

**DOI:** 10.14202/vetworld.2024.448-461

**Published:** 2024-02-23

**Authors:** Libaihe Jing, Jie Xu, Jiao Cai, Shan Huang, Xinyu Qiao, Fengqi Wan

**Affiliations:** 1 Cuiying Biomedical Research Center, Lanzhou University Second Hospital, Lanzhou, 730030, Gansu, China; 2 Department of Reproductive Medicine, Lanzhou University Second Hospital, Lanzhou, 730030, Gansu, China; 3 The Second School of Clinical Medicine, Lanzhou University, Lanzhou, 730030, Gansu, China

**Keywords:** artiodactyl, bone functional adaptation, calcaneus, captivity, morphological variation, *Saiga tatarica*

## Abstract

**Background and Aim:**

Captivity alters the locomotor behavior of wild artiodactyls and affects the mechanical loading of the calcaneus; however, the resulting adaptive changes in calcaneus morphology have not been sufficiently studied to date. This study aimed to investigate the morphological and mechanical adaptive variations in the calcaneus of *Saiga tatarica* to understand further the functional adaptation of the calcaneus in wild artiodactyl to captivity.

**Materials and Methods:**

Paired calcanei from autopsy samples of six captive wild artiodactyls (*S. tatarica*) and six domesticated artiodactyls (*Ovis aries*) were divided into skeletally immature and mature groups using X-ray evaluation of growth plate closure. High-resolution microcomputed tomography revealed a calcaneal diaphyseal cross-section. The mechanical and nanomorphological characteristics of the trabecular bone were determined by atomic force microscopy.

**Results:**

The percent cortical bone area (%CA), cortical thickness ratio (CTR), and Young’s modulus (*E*) differed between species in the immature groups but not in the mature groups. *S. tatarica* had significantly higher growth rates for %CA, CTR, and *E* in the mid-shaft than *O. aries* (p < 0.05).

**Conclusion:**

The calcaneus morphology of *S. tatarica* converges with that of domesticated *O. aries* during ontogeny. These results indicate that the calcaneus of wild artiodactyls can undergo potentially transitional changes during the short-term adaptation to captivity. The above parameters can be preliminarily identified as morphological signs of functional bone adaptation in artiodactyls.

## Introduction

Habitual locomotor patterns determined by habitat affect the loads placed on limb bones during locomotion and influence variations in bone morphology [1, 2]. Thus, such bone morphology variations are considered to be adaptive responses to loading conditions that directly reflect lifestyle-related functional bone adaptation [1, 3]. The calcaneus is the main weight-bearing bone in the hock joint of artiodactyls and presents a habitually loaded cantilevered beam-like structure during bending. In combination with beam theory and Wolff’s law, different load conditions tend to induce variations in the calcaneal diaphysis and regional trabecular bone morphology [4–6]. Therefore, calcaneus has been recommended as a model for investigating bone functional adaptation under environmental mechanical stress [7]. Genetically derived bone morphological traits can be altered by bone remodeling to adapt to environmental stress conditions [8].

It has been proposed that the skeletal physiological response to mechanical stimuli is related to loading patterns determined by different habitats; therefore, habitat has an important effect on bone developmental patterns. In Artiodactyla, differences between captive and natural habitats induce variations in the morphological response of the calcaneus to locomotor behavior [1, 8]. Within the same habitats, calcaneal adaptive architectures of captive wild and domesticated artiodactyls may be correlated. Reduced mobility in captivity may lead to plastic (non-heritable) changes in the calcaneus of wild artiodactyls. A potential hypothesis is that the morphological patterns of the calcaneus of captive wild artiodactyls would undergo transitional variation. However, few studies have investigated the calcaneal morphology of captive wild artiodactyls, with only limited research performed in wild *Odocoileus hemionus hemionus* and domesticated *Ovis aries* [7, 9]. Although the restoration and reintroduction of captive wild animals into the wild is a key issue in the field of species conservation, their adaptability to the habitat has been largely overlooked. Observational data on captive wild animals with little habitual extreme locomotor behavior show that they are often injured during high-intensity activity when released into the wild due to insufficient limb bone strength [10, 11]. *Saiga tatarica* is a rare and endangered wild animal, and it is critical to uncover their morphological adaptability in captivity for the protection of this species.

*S. tatarica* in a semi-free-ranging enclosure are likely to have experienced less habitual extreme locomotor behavior, such as running or jumping, than those in the wild, which could cause differences in the habitual loading of the calcaneus. This differential loading is expected to induce calcaneal morphology that is more suited to lower strains and bending loads produced from their habitual activity compared to wild artiodactyl, which experience more extreme habitual loads and activity levels [4]. Therefore, the calcaneus of *S. tatarica* may be suitable for studying functional bone adaptation in captivity.

In captivity, the pattern of transitional variation in the calcaneal form of wild artiodactyls has not been established. The effect of reduced mobility in captivity on the calcaneus of wild *S. tatarica* warrants further investigation. The aim of the present study was to reveal the morphological and mechanical changes in the calcaneus of wild *S. tatarica* during short-term adaptation to captivity. To further understand the functional adaptation of the calcaneus to captivity in wild artiodactyl.

The present study investigated the cross-sectional geometry, trabecular nanostructure, and trabecular mechanical properties of the calcaneus in captive artiodactyls by combining micro computed tomography (micro-CT)-derived geometric morphometrics and atomic force microscopy (AFM).

## Materials and Methods

### Ethical approval

The specimens used in this study were from deceased individuals donated by others, none of the animals were specifically sacrificed for this study. Hence, ethical approval was not needed.

### Study period and location

The sampling was conducted in the spring of 2020 (February to May) at Gansu Protection Center of Endangered Animals (37.9°N, 102.9°E, elevation = 1766 m above sea level) and a small slaughterhouse in Wuwei City, Gansu Province.

### Sample preparation

**Figure-1:**
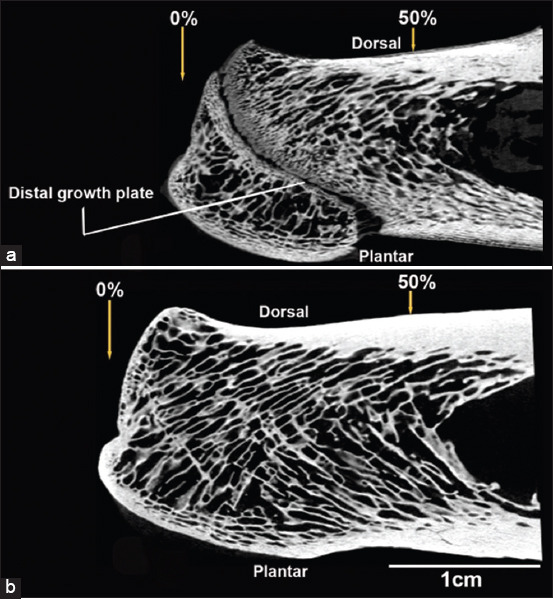
Representative micro-computed tomography (micro-CT) images of the medial-to-lateral view of the left *Saiga tatarica* calcanei: (a) immature specimen, and (a) mature specimen. 0% is located at the distal-dorsal “notch” and 50% is located at the mid-shaft location of the calcaneal diaphysis. (a) An “immature” specimen with an unclosed distal growth plate.

Samples from paired calcanei of six captive wild artiodactyls (*S. tatarica*) and six domesticated artiodactyls (*O. aries*) were obtained during autopsy. *S. tatarica*, representing captive wild artiodactyls, was recruited from the zoo at Gansu Protection Center of Endangered Animals (37.9°N, 102.9°E, elevation = 1766 m above sea level), which is one of the most important resource centers for *S. tatarica* with an area of 850,000 ha. *S. tatarica* bone specimens came from individuals who died of natural causes and dystocia. *O. aries*, representing domesticated artiodactyls, were used as controls. Bone specimens of *O. aries* were obtained from paddocks where they were grown for human consumption. None of the animals were specifically sacrificed for this study. This study was conducted according to the guidelines for the care and use of animals [12]. The sex was not an inclusion criterion for this study. All study specimens were obtained from animals that did not show any signs of disease affecting their bones or lameness. Bone maturity was determined by the degree of fusion of the distal epiphysis using X-ray as follows: Immature bone (3–6 [mean: 3.75 ± 1.54] months, *S. tatarica*, n = 3; *O. aries*, n = 3), and mature bone (20–48 [mean: 31.7 ± 9.26] months, *S. tatarica*, n = 3; *O. aries*, n = 3) (Figure-1 and Table-1).

**Table-1:** Specimens.

Taxon	Habitat	Body mass (kg) Mean (SD)	Age group	n
*Saiga tatarica*	Captive-wild	13.33 (0.93)	Immature	3
		58.57 (5.45)	Mature*	3
*Ovis aries*	Domesticated	14.70 (1.93)	Immature	3
		40.56 (4.28)	Mature*	3

* Specimens were considered “mature” if the distal growth plate was closed

**Figure-2:**
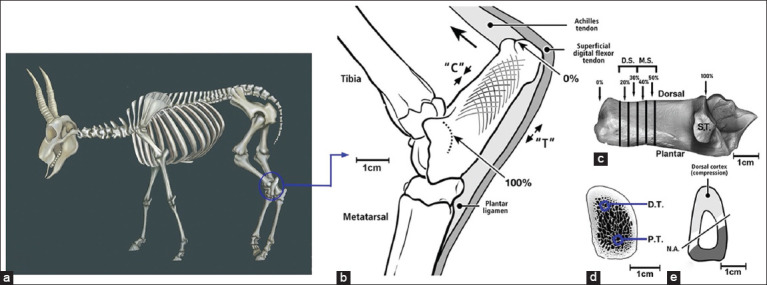
Bone orientation and positioning in the *Saiga tatarica* (a) diagrammatic skeleton of the *S. tatarica* Jing *et al*. [1]. (b) Lateral-to-medial view of the hock of a skeletally mature *S. tatarica* showing the calcaneus shaft length, and other associated tissues. The stylized trabecular patterns are determined based on a medial-to-lateral roentgenogram Skedros *et al*. [7, 13]. Calcaneus shaft length from the distal-dorsal “notch” at 0% to the projected location of the contour formed by the talus-calcaneus articular surface at 100%. The large dorsal directed arrow shows the direction of force exerted by the Achilles tendon during mid-stance, the dorsal side is loaded in compression (“C”) and the plantar side is loaded in tension (“T”) Skedros *et al*. [9], Su *et al*. [14]. (c) Medial-to-lateral view of a left adult *S. tatarica* calcaneus. MS: Mid-shaft (40% and 50% segments); DS: Distal shaft (20% and 30% segments); ST: Sustentaculum talus. (d) The cross-section is from a 50% section and shows the region from which the atomic force microscopy (AFM) scans (DT: Dorsal trabecular tracts, PT: Plantar trabecular tracts) were taken and (e) the approximate location of the neutral axis (NA). According to the actual size of the mature *S. tatarica* calcaneus, the proportion of hock (b) and cross section of calcaneus (e) were modified from Skedros *et al*. [7, 9].

Adherent soft tissues were removed prior to data acquisition. Bones were manually oriented and positioned according to the percentage length as described by Skedros *et al*. [13] (Figure-2) [1, 7, 9, 13, 14]. The diaphyseal length of each bone was measured using a digital Vernier caliper (Mitutoyo, Japan), and the error was controlled to within 1000ths of a millimeter. Segments from the distal shaft (DS) of the calcaneus (means of 20% and 30% locations) and mid-shaft (MS) of the calcaneus (means of 40% and 50% locations) were analyzed. The diaphysis axis was defined as the axial direction, and the radial direction was defined as the direction perpendicular to the axis (Figure-S1).

**Figure-S1:**
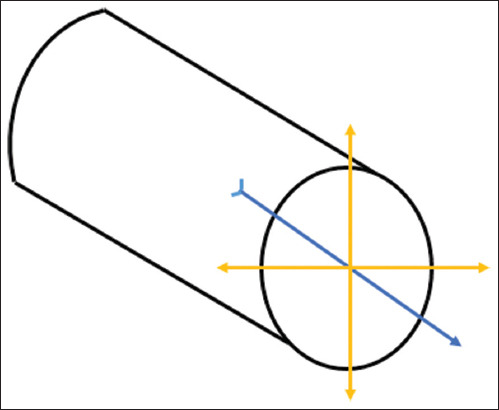
Diagram of the orientation of the artiodactyl calcaneus. Yellow double arrows represent radial direction; blue arrow represents the axial direction.

### Micro-CT scanning of the bone

Three left calcanei from each age group of both species were scanned at a 35-micron resolution (90 kVp, 0.08 mA, and exposure time of 500 ms) using a high-resolution micro-CT scanner (Pingseng Healthcare Inc., NEMO NMC-100, Shanghai, China). The resulting voxel size was 0.0293 × 0.0293 × 0.06 mm for all scans. Imaging data were exported as 16-bit 1024 1024 for three-dimensional (3D). Each percent diaphysis location segment was divided into 39 slices with a thickness of 0.06 mm. A single micro-CT slice at the prerecorded distance for each percent diaphysis location was exported as an 8-bit TIFF image format to obtain cross-sectional geometry measurements of the cortical bone. We then obtained cross-sectional geometric data using the ITK 4.5.2 software (https://itk.org/download/). Data were measured and averaged for each section. Geometric morphometry is the most direct and important criterion in the comparative study of bone structure. The diaphyseal cross-sectional properties, on which we focus here, are the following geometric parameters: (1) Section modulus (*Z*), which is approximately proportional to the bending or torsional moment that characterizes the cross-sectional strength of the bone [15], is a good indicator of a bone’s cross-sectional mechanical performance; (2) cortical thickness ratio (CTR, the dorsal cortical thickness/the plantar cortical thickness), which represents asymmetry of the cortical bone distribution between dorsal and plantar surfaces as well as a more defined direction of greatest bending rigidity; (3) the ratio of principal moments of area (Imax/Imin), which is an estimation of cross-sectional shape and bending rigidity; the larger the cross-sectional Imax/Imin ratio deviates from one, the greater the departure from circularity; and (4) percent cortical bone area (%CA, cortical area [CA]/total cross-sectional area), which provides an estimate of axial compressive or tensile strength. Calcaneal diaphyseal cross-sectional properties were measured at the MS (means of 40% and 50% locations) and DS locations (means of 20% and 30% locations).

### AFM imaging of the bone

Three additional right (contralateral) calcanei obtained from the same bone pairs used in the micro-CT analyses were used. The calcaneal diaphysis was sliced using a water-cooled diamond blade saw (Exakt, Germany). Serial sectioning of the calcaneal diaphysis was performed using a hard tissue microtome (Leica SP1600, Hexagon Co., Germany) (Figure-S2), and the calcaneal diaphysis was sectioned into 2 mm thick slices from 20% to 50% of the shaft length. Thin sections (n = 1/location) were ground (600 grit paper) and buffed on a lapidary wheel to obtain an overall thickness of 1.5 mm. A transversely cut segment was obtained from the 20%, 30%, 40%, and 50% shaft locations of each of the three bones in each age group of the two species. The specimens were ultrasonically cleaned in distilled water for 5 min and then dried naturally at 15–25°C. Sections were fixed on microscope slides for AFM imaging. To ensure the validity of the inter-specimen comparisons, all the specimens were prepared and analyzed similarly.

**Figure-S2:**
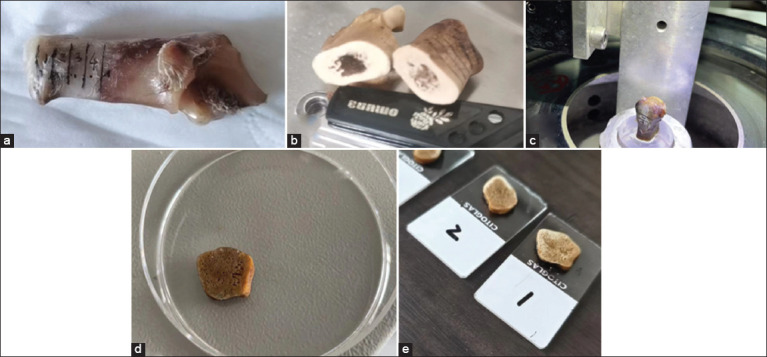
Serial sectioning and preparation of bone samples. (a) Calcaneus and its regional division, (b) excision of calcaneal diaphysis, (c) fixed the calcaneal diaphysis on the hard tissue microtome (LEICA SP1600), (d) slicing of calcaneal diaphysis, (e) calcaneal diaphyseal cross-section.

Each slide containing a bone sample was placed horizontally and glued to the sample disk (Figure-S3). A biological AFM (BioAFM; NanoWizard III, Bruker, USA) was used under ambient conditions to analyze the apparent topography and force spectroscopy results. AFM was operated in contact mode using a silicon SPM sensor (Nanoworld Instruments, Inc.) with a cantilever length of 125 μm, force constant of 42 N/m, resonance frequency of 320 kHz, and tip width of 30 μm. The scanning sizes were 10 × 10 μm^2^ and 1 × 1 μm^2^, and a line rate of 0.9 Hz was used. Force–distance curves were generated when the probe contacted the osseous tissue. The structure, morphology, and mechanical properties of the samples were measured at 5 μm/s. Ten random sites from the trabecular bone within the dorsal and plantar regions were selected for each sample, and each site was measured 10 times. The intratrabecular “tract” (dorsal and plantar) is also referred to as “regions” (compression and tension). The force–distance curves and Young’s modulus (*E*) were analyzed using the modified Hertz contact model. The Poisson’s ratio was 0.3. The root-mean-square surface roughness (RMS roughness) and grain sizes were estimated from the topography images using the JPKSPM data processing software (Software Version 6.003/2019, https://sourceforge.net/projects/gwyddion/files/pygtk-win32). We estimated the grain size by measuring the maximum dimension of the grains. Nanomorphology and mechanical properties of the trabecular bone were obtained in the dorsal “compression” and plantar “tension” regions of the MS (means of 40% and 50% locations) and DS (means of 20% and 30% locations) locations.

**Figure-S3:**
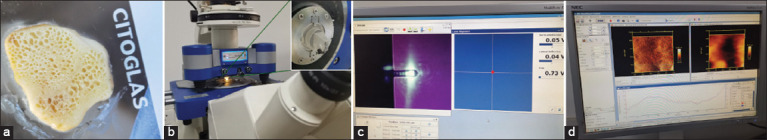
Fixation and scanning of bone samples. (a) Fixation of sample, (b) installation of probe, (c) calibration of the laser, and (d) computer imaging by ITK 4.5.2 software.

### Statistical analysis

Origin Lab (version 8, https://www.originlab.com) was used for data analysis and curve fitting. The images were processed and rendered using Adobe Photoshop 2021 software (https://www.adobe.com/products/photoshop.html). SPSS version 22 (https://spss-64bits.en.softonic.com) was used for statistical analyses. The Shapiro–Wilk test was used to determine the normality of the data distributions. Student’s t-test was used in the case of a normal distribution; otherwise, the Mann–Whitney *U*-test was used. Calcaneal cross-sectional parameters (Imax/Imin, %CA, CTR, and *Z*) of different locations of *S. tatarica* and *O. aries* were log10 transformed and compared. Comparisons between locations of different species in each age group, analyses of differences between age groups in the same species, and analyses of differences between species in the same age group were performed. We compared trabecular parameters (GS, RMS roughness, and *E*) of the entire calcaneal diaphysis of *S. tatarica* and *O. aries* and of different regions in the calcaneus. The radial (dorsal–plantar plane) and axial (along the calcaneal diaphysis) parameters of *S. tatarica* and *O. aries* were pooled to form sets of the whole calcaneus in each age group. Regional data sets were obtained by grouping *S. tatarica* or *O. aries* trabecular parameters that formed the same region but were from different samples of the same age group. Differences in the entire calcaneus were compared between two age groups in the same species and between species in the same age group. Comparisons between the regions of different species in each diaphyseal location by age group, differences in regional parameters in the same species between different age groups, and differences in regional parameters in the same species between different age groups were also performed. Data are expressed as means and standard deviations (SD). Differences were considered statistically significant at p < 0.05. Correlations between the micro-CT and AFM data were not determined because they were not obtained from the same bones.

## Results

### Morphology of the calcaneal diaphyseal cross-sectional cortical bone and diaphyseal shape

Cross-sectional geometric parameters (Imax/Imin, %CA, CTR, *Z*) are credible indicators of cross-sectional shape and robustness [7, 14]. Adaptive variations in the morphology of the calcaneus were evaluated using microCT through the analysis of the calcaneal cross-sectional geometry Table-2 lists the mean values, SDs, and exact p-values for the morphometric variables of the calcaneal shape and cross-sectional cortical bones in the DS and MS of the artiodactyl calcaneus.

**Table-2:** Cortical bone analysis for different locations of the calcaneal diaphysis by species (mean [SD]).

Parameters	Locations	Species	Immature bones (saiga antelope: n = 3; sheep: n = 3)	Mature bones (saiga antelope: n = 3; sheep: n = 3)	Difference between age groups (p-value)
Imax/Imin	Distal shaft	Saiga	0.397 (0.020)^#,^*	0.497 (0.078)^#,^*	**0.033**
		Sheep	0.096 (0.043)*	0.119 (0.025)*	0.620
	Mid-shaft	Saiga	0.789 (0.058)^#^	0.965 (0.050)^#^	**0.012**
		Sheep	0.227 (0.041)	0.314 (0.115)	**0.024**
%CA	Distal shaft	Saiga	1.187 (0.036)^#,^*	1.253 (0.048)*	**0.047**
		Sheep	1.238 (0.032)*	1.262 (0.050)*	0.076
	Mid-shaft	Saiga	1.568 (0.041)^#^	1.666 (0.029)	**0.021**
		Sheep	1.673 (0.046)	1.683 (0.035)	0.651
CTR	Distal shaft	Saiga	−0.443 (0.052)*	−0.192 (0.129)*	**0.004**
		Sheep	−0.460 (0.073)*	−0.224 (0.078)*	**0.012**
	Mid-shaft	Saiga	−0.174 (0.204)^#^	0.259 (0.047)	**0.000**
		Sheep	−0.111 (0.043)	0.220 (0.106)	**0.007**
Z	Distal shaft	Saiga	2.335 (0.111)*	2.569 (0.163)^#,^*	**0.027**
		Sheep	2.284 (0.061)*	2.461 (0.141)*	**0.030**
	Mid-shaft	Saiga	2.474 (0.089)	2.725 (0.123)^#^	**0.009**
		Sheep	2.391 (0.076)	2.590 (0.134)	**0.036**

Paired samples t-test (two-tailed): Contrast between the distal shaft (means for 20% and 30% locations) and mid-shaft (means for the 40% and 50% locations) properties by species.

* Significantly different between the distal and mid-shafts of the artiodactyl calcaneus in the same age group; p < 0.05.

# Significantly different between saiga antelope and sheep calcaneal parameters in the same age group; p *<* 0.05. p-values (between age groups) < 0.05 are shown in bold. All data were log10 transformed. SD=Standard deviation, CTR=Cortical thickness ratio

#### Comparisons between species in the same age group

Imax/Imin values in the distal and MS of the calcaneal diaphysis of *S. tatarica* were significantly higher than those of *O. aries* in both the immature and mature groups (p < 0.05). The diaphyseal cross-section of *S. tatarica* calcaneus was more oval/oblong along the dorsal/plantar plane than along the dorsal plane. *S. tatarica* may possess a relatively high dorsal–plantar bending rigidity.

In the immature groups, *S. tatarica* presented significantly lower %CA values in both diaphyseal locations than *O. aries* (p < 0.05). However, there was no significant difference in %CA values between the mature groups at either diaphyseal location (DS: p = 0.467; MS: p = 0.499).

There was a significant difference in CTR between *S. tatarica* and *O. aries* in the MS of the calcaneus in the immature groups; *S. tatarica* had a significantly lower CTR than that of *O. aries* (p < 0.05). *S. tatarica* had higher CTR values than *O. aries*, but no significant differences were found in either the DS or MS of the calcaneal diaphysis (DS: p = 0.077; MS: p = 0.419, respectively).

In both age groups, *Z* values of *S. tatarica* were higher than those of *O. aries* at both diaphyseal locations, with significant differences observed in the mature groups (p < 0.05).

#### Comparisons between diaphyseal locations in the same age group by species

The MS of the calcaneal diaphysis showed significantly higher values for all parameters compared with those of the DS in individuals of each age group in both species (Imax/Imin, CTR, and %CA, p < 0.01; *Z*, p < 0.05). The diaphyseal cross-sectional shapes of the distal and MSs of the cantilevered beam-like bone (artiodactyl calcaneus) differed because of the different loading conditions along the calcaneal diaphysis.

#### Comparisons between age groups by species

The mature groups of both species presented significantly higher values for all parameters than the immature groups, which was caused by increased bone strength during growth and development.

### Trabecular nanotopography and mechanical properties of the whole calcaneal diaphysis

AFM enables the nanoscale structural and mechanical characterization of trabecular bone, including assessment of grain size, RMS roughness, and *E*. Table-3 lists the mean values and SDs for grain size, RMS roughness, and *E* to assess changes in the entire calcaneus based on nanoscale characterizations. Representative AFM images of regions of interest for some cross-sections from each species are shown in an AFM topography image of the trabecular bone as shown in Figure-3. These images are representative of those acquired from various areas of the sample and consistently depict the observed characteristics. The observed bony surfaces represent a continuous phase, but have a distinct granular structure (AFM deflection image, Figure-3).

**Table-3:** Morphological and mechanical parameters of trabeculae for the entire calcaneus shaft (mean [SD]).

Parameters	Species	Immature bones (saiga antelope: n=3; sheep: n=3)	Mature bones (saiga antelope: n=3; sheep: n=3)	Difference between age groups (p-value)
Grain sizes (mm)	Saiga	0.29 (0.07)	0.33 (0.09)	**0.019**
	Sheep	0.23 (0.04)	0.28 (0.08)	**0.032**
RMS roughness (nm)	Saiga	139.40 (80.57)^#^	127.21 (52.91)^#^	0.375
	Sheep	225.84 (79.99)	154.60 (92.58)	**0.028**
Young’s modulus (GPa)	Saiga	2.91 (1.93)	3.63 (1.60)	**0.012**
	Sheep	3.25 (1.51)	3.48 (2.20)	0.258

The means and standard deviations (SD) are shown. p-values (between age groups) < 0.05 are shown in bold.

# Significantly different between saiga antelope and sheep in the same age group; p < 0.05

**Figure-3:**
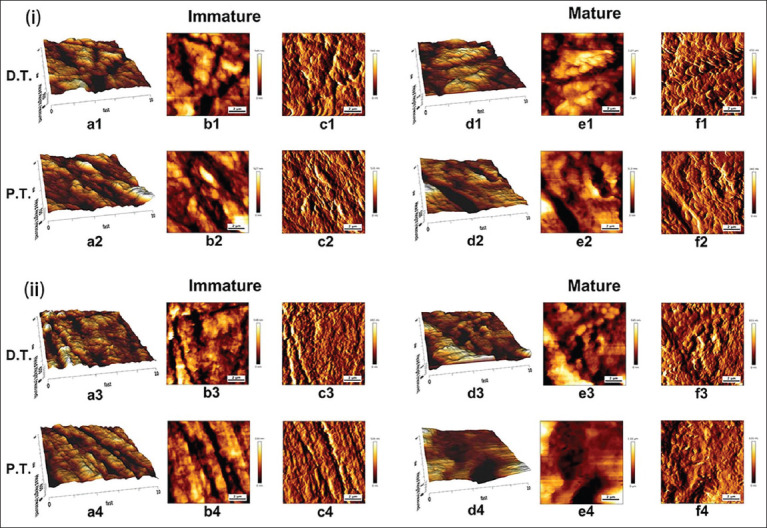
Representative atomic force microscopy (AFM) images of calcaneal transverse sections from (i) captive wild (*Saiga tatarica*) and (ii) domesticated artiodactyl (*Ovis aries*). The images show the distal-most portion of the segment. (a1–a4) and (d1–d4): Three-dimensional topographic AFM images of immature and mature calcanei. (b1–b4) and (e1–e4): AFM height images of immature and mature calcanei. (c1–c4) and (f1–f4): AFM deflection images of immature and mature calcanei. Each graph shows a 10 (Fast) × 10 (Slow) mm area. DT=Dorsal trabeculae, PT=Plantar trabeculae.

#### Comparisons between species in the same age group

Analysis of the AFM images revealed that *S. tatarica* grains were larger than *O. aries* grains in both age groups; however, the differences were not significant (immature: p = 0.151; mature: p = 0.098). In each age group, RMS roughness in *O. aries* was significantly higher than that in *S. tatarica* (p < 0.05). In the immature groups, *E* of *S. tatarica* was lower than that of *O. aries*, whereas the reverse trend was observed in mature groups (p > 0.05).

#### Comparisons between age groups by species

**Figure-4:**
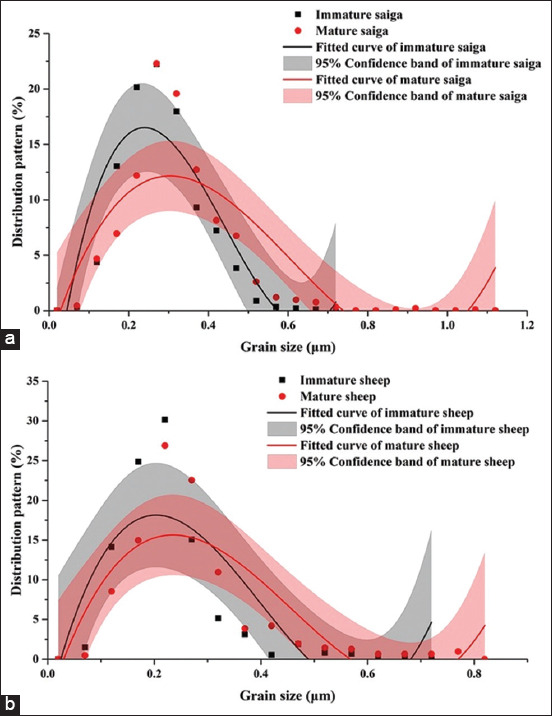
Fitted curves for grain size distribution in the whole calcaneal diaphysis of (a) Captive wild (*Saiga tatarica*) and (b) domesticated artiodactyl (*Ovis aries*) (black curves are immature individuals, red curves are mature individuals). Curve shape denotes the grain size distribution. Fitted curves for grain size distribution that are shifted more to the right represent increased average grain size. The peak indicates the highest proportion of grains with a certain size.

Significant differences were observed in grain size between the two age groups in both species (Table-3 and Figure-4). The grains of mature bones over the calcaneal diaphysis in both species were significantly larger than those of immature bones on average (p < 0.05). Changes in RMS roughness revealed age-related decreases, and lower RMS roughness values were observed in mature groups of the two species compared with those in immature groups; however, the difference was only significant in *O. aries* (p < 0.05). There was a significant difference in *E* in *S. tatarica* between the two age groups, where *E* was significantly higher in the mature group compared with that in the immature group (p < 0.05).

### Trabecular nanotopography and mechanical properties of localized regions of the calcaneus

Table-4 shows the mean values and SD of the surface morphological and mechanical parameters of the trabecular nanostructures in the two regions of both species. Dorsal and plantar regional data from the DS and dorsal and plantar regional data from the MS were separately collected for analysis. Subsequently, we compared the localized diaphyseal regions (dorsal and plantar) between the two species.

**Table-4:** Trabecular bone analysis for different regions in the distal and mid-shafts of the calcaneus (mean [SD]).

Parameters	Region	Species	Immature bones (saiga antelope: n = 3; sheep: n= 3)	Mature bones (saiga antelope: n = 3; sheep: n = 3)	Difference between age groups (p-value)
Distal shaft					
Grain sizes (μm)	Dorsal	Saiga	0.369 (0.077)^#,^*,**	0.399 (0.042)^#,^*,**	**0.041**
		Sheep	0.189 (0.018)*	0.258 (0.073)**	**0.000**
	Plantar	Saiga	0.257 (0.056)	0.279 (0.014)**	**0.009**
		Sheep	0.246 (0.047)	0.248 (0.018)	0.885
RMS roughness (nm)	Dorsal	Saiga	85.11 (38.47)^#,^*,**	82.78 (64.92)^#,^*,**	0.461
		Sheep	332.49 (150.49)*,**	146.94 (95.91)	**0.006**
	Plantar	Saiga	138.63 (27.73)^#^	124.07 (44.84)^#^	**0.047**
		Sheep	236.83 (45.92)**	159.41 (29.94)	**0.009**
Young’s modulus (GPa)	Dorsal	Saiga	2.99 (1.65)**	3.64 (2.83)*	**0.002**
		Sheep	2.85 (1.68)	3.61 (1.30)	**0.013**
	Plantar	Saiga	2.72 (1.25)^#,^**	3.02 (1.98)**	**0.000**
		Sheep	3.67 (0.67)	3.36 (2.64)	0.773
Mid-shaft					
Grain sizes (μm)	Dorsal	Saiga	0.234 (0.016)^#,^*	0.278 (0.065)^#,^*	**0.000**
		Sheep	0.196 (0.046)*	0.341 (0.124)*	**0.000**
	Plantar	Saiga	0.276 (0.017)	0.362 (0.069)^#^	**0.000**
		Sheep	0.251 (0.04)	0.279 (0.086)	**0.021**
RMS roughness (nm)	Dorsal	Saiga	181.58 (72.02)	170.06 (88.21)*	**0.042**
		Sheep	192.61 (42.23)*	136.78 (47.90)*	**0.009**
	Plantar	Saiga	154.83 (98.75)	128.11 (45.84)^#^	**0.000**
		Sheep	141.23 (53.01)	167.01 (68.19)	0.053
Young’s modulus (Gpa)	Dorsal	Saiga	2.31 (0.98)^#,^*	3.59 (1.29)*	**0.000**
		Sheep	3.28 (0.71)	3.37 (2.53)	0.296
	Plantar	Saiga	3.43 (1.35)	4.13 (2.71)	**0.041**
		Sheep	3.31 (1.18)	3.58 (1.24)	0.623

The means and standard deviations (SD) are shown. p-values (between age groups) < 0.05 are shown in bold. Dorsal: Dorsal “compression” region; plantar: Plantar “tension” region.

# Significantly different between saiga antelope and sheep in the same age group; p < 0.05.

* Significantly different between dorsal “compression” and plantar “tension” regions in the same age group; p < 0.05.

** Significantly different between the distal and mid-shaft locations of diaphysis in the same group; p < 0.05

#### Comparisons between species in the same region

In both the distal and MS, grain sizes in the dorsal region of *S. tatarica* differed significantly from those of *O. aries*, with the same trend (*S. tatarica* > *O. aries*, p < 0.05). No significant differences between the species were observed in grain size in the plantar regions of either the distal or mid-shaftMS (DSdistal shaft: p = 0.214, mid-shaftMS: p = 0.141). In mature groups, grain sizes in the dorsal region of both diaphyseal locations of the calcaneus in *S. tatarica* were significantly different from those in *O. aries*; grain sizes in the dorsal region of the DS in *S. tatarica* were significantly larger than those in *O. aries* (p < 0.05), whereas the trend was reversed in the MS (*S. tatarica* < *O. aries*, p < 0.05); grains in the plantar region in the MS of *S. tatarica* were significantly larger than those in *O. aries* (p < 0.05).

RMS roughness in both regions in the DS of *S. tatarica* was significantly lower than that of *O. aries* (p < 0.05) in the immature groups. There were no significant differences in RMS roughness between species in immature individuals in the MS (dorsal: p = 0.215, plantar: p = 0.198) in either region. The mature group presented the same differences between species in the dorsal and plantar regions in the DS (*S. tatarica* < *O. aries*, p < 0.05) as the immature group. In the MS, there was a significant difference in RMS roughness between mature *S. tatarica* and mature *O. aries* in the plantar region (p < 0.01).

A significant difference in *E* between species was observed in the plantar region of the DS in the immature groups (*S. tatarica* < *O. aries*, p < 0.05). There was a significant difference in *E* between species in the dorsal region (*S. tatarica* < *O. aries*, p < 0.05) in the MS. No significant difference was observed in *E* between mature *S. tatarica* and *O. aries* in either of the two regions for any diaphyseal location.

### Dorsal and plantar region comparisons by species

In *S. tatarica* immature groups, grain sizes in the dorsal region of the DS were significantly larger than those in the plantar region; this trend was reversed in the MS (dorsal < plantar, p < 0.01). In both diaphyseal locations, grains in the dorsal region were significantly smaller than those in the plantar region in immature *O. aries* (p < 0.05). Mature *S. tatarica* were identical to immature individuals in terms of grain size differences between the dorsal and plantar regions in both diaphyseal locations (DS: dorsal > plantar, p < 0.05; MS: dorsal < plantar, p < 0.05). Grain sizes were significantly larger in the dorsal region than in the plantar region in the MS of mature *O. aries* (p < 0.05).

There were significant differences in RMS roughness between the dorsal and plantar in the DS in both species in the immature groups; however, reverse trends were observed (*S. tatarica*: Dorsal < plantar, *O. aries*: Dorsal > plantar, p < 0.05). There was a significant difference in RMS roughness between the two regions in *O. aries* in the immature group in the MS; the dorsal region presented a significantly higher value than the plantar region (p < 0.05). In the DS, RMS roughness in mature *S. tatarica* was significantly lower in the dorsal region than in the plantar region (p < 0.05), whereas there was no significant difference in RMS roughness between the dorsal and plantar regions in mature *O. aries* (p = 0.512). There were significant regional differences in RMS roughness in the MS of the mature groups in both species; however, the trends were reversed for *S. tatarica* and *O. aries*: The RMS roughness in *S. tatarica* in the dorsal region was significantly higher than that in the plantar region (p < 0.05), and the RMS roughness in *O. aries* in the dorsal region was significantly lower than that in the plantar region (p < 0.05).

There was a significant difference in *E* between the two regions in the MS of *S. tatarica* calcaneus in the immature groups (dorsal < plantar, p < 0.05). In mature *S. tatarica*, *E* was significantly greater in the dorsal region than in the plantar region in the DS (p < 0.05), whereas it was significantly lower in the dorsal region than in the plantar region in the MS (p < 0.05). Mature *O. aries* and *S. tatarica* presented the same trend of change in radial directions of the two diaphyseal locations, but there were no significant differences in *E* values between the dorsal and plantar regions in *O. aries*.

#### Distal and MS comparisons by species of the calcaneus

In the immature groups, grains in the DS were significantly larger than those in the MS and were found only in the dorsal region of *S. tatarica* calcaneus (p < 0.05). No significant differences in grain size were observed between the two diaphyseal locations in the dorsal or plantar regions of immature *O. aries* calcaneus (dorsal: p = 0.125; plantar: p = 0.098). In mature *S. tatarica* calcaneus, grains in the dorsal region of the DS were significantly larger than those in the MS (p < 0.01); however, the trend reversed in the plantar region (DS < MS, p < 0.05). In mature *O. aries*, grains were significantly smaller in the dorsal region of the DS than in the MS region (p < 0.05). No significant difference in grain size was observed between the plantar MS and plantar DS in mature *O. aries* (p = 0.246).

In immature *S. tatarica*, RMS roughness was significantly lower in the dorsal DS than in the dorsal MS in the dorsal region of the calcaneus. No significant difference in RMS roughness was found between locations in the plantar region of immature *S. tatarica* calcaneus (p = 0.215). RMS roughness values were significantly higher in the dorsal and plantar regions of the DS than in the corresponding regions of the MS in immature *O. aries* (p < 0.01). Mature and immature *S. tatarica* were similar in that there was a significant difference only between the distal and MS of the dorsal region (DS < MS, p < 0.01). No significant differences in RMS roughness were observed between the two diaphyseal locations in either the dorsal or the plantar regions in mature *O. aries* (dorsal: p = 0.278; plantar: p = 0.199).

In the immature groups, significant differences in *E* were observed between the diaphyseal locations in both the dorsal and plantar regions of *S. tatarica* calcaneus. However, changes in *E* values in different regions followed opposite trend in axial direction (dorsal DS > dorsal MS, plantar DS < plantar MS, p < 0.05). No significant differences in *E* were observed between diaphyseal locations in either region of immature *O. aries* (dorsal: p = 0.357; plantar: p = 0.179). A significant difference in *E* was observed between the two diaphyseal locations in the plantar region of *S. tatarica* calcaneus (DS < MS, p < 0.05). *E* was greater in the dorsal DS of mature *S. tatarica* calcaneus than in the dorsal MS; however, the difference was not significant (p = 0.245). *E* values in the dorsal regions of the DS were higher than those in the corresponding regions in the MS, and *E* values in the plantar regions of the DS were lower than those in the corresponding regions in the MS in mature individuals. In other words, there were significant differences in the *E* value of mature *S. tatarica* plantar regions between the two diaphyseal locations (p < 0.05). However, no significant difference was observed in *E* values of the dorsal and plantar regions of mature *O. aries* between the two diaphyseal locations (dorsal: p = 0.387; plantar: p = 0.337).

#### Comparisons between age groups by species

In both diaphyseal locations, grain size and *E* of both species in the two regions were lower in the immature group than in the mature group, with the exception of the plantar region in the DS of *O. aries* (immature > mature, p > 0.05). The RMS roughness of both species in the two regions was higher in the immature groups than in the mature groups, with the exception of the plantar region in the MS of *O. aries* (immature < mature, p > 0.05).

## Discussion

This study investigated the effects of a captive environment on bone strength and morphology indices in the calcaneus of captive wild artiodactyls. The calcaneus is a short, cantilevered beam with longitudinal compression and tension strains predominating in opposing dorsal and plantar aspects of the bone and is the major weight-bearing bone in the hock joint of artiodactyls [4, 6]. It is used as a model for interpreting bone adaptation to habitual loads. While strain gauge studies on the artiodactyl calcaneus reported predominant loading of the cortical bone, the role of cancellous bone in response to stress should not be ignored, especially the effects of changes in the nanostructure of the trabecular bone on mechanical behaviors [6]. These subtle changes are likely to reveal the underlying mechanisms of adaptive bone remodeling. The possibility that these changes reflect “lifestyle-related functional adaptations” is supported by data in the present study showing that the diaphyseal cross-sectional cortical bone area, cortical bone thickness, and nano-mechanical properties (i.e., *E*) of the calcaneal bone markedly differ in immature captive wild and domesticated artiodactyl individuals, whereas these characteristics converge during adulthood. To the best of our knowledge, this is the first study to reveal the functional adaptation of the calcaneus during ontogeny in captive wild artiodactyls.

### Lifestyle-related functional adaptations morphological signals in the calcaneal cortical bone

Evolutionarily, cursorial species have greater cortical bone asymmetry and cross-sectional flattening, presenting a greater modulus and CA [9, 16, 17]. These typical features reflect extreme habitual loads and high activity levels in the wild, which require increased bone strength [7]. Studies on functional adaptation of bone in wild *O. hemionus hemionus* and domesticated *O. aries* suggest that artiodactyls in different stress habitats can be differentiated using the geometry of their calcaneal diaphyseal cross-section [4]. Imax/Imin in wild immature *O. hemionus hemionus* is significantly higher than that in domesticated immature *O. aries*, and the growth rate of Imax/Imin in adulthood is faster than that in domesticated *O. aries* [7]. Similarly, at an early age, our results showed significantly greater Imax/Imin values in *S. tatarica* than in *O. aries*. With development, *S. tatarica* presented a faster growth rate of Imax/Imin than that in *O. aries* (DS: mature *S. tatarica* was 25.2% higher than immature *S. tatarica*, mature *O. aries* was 23.9% higher than immature *O. aries*, p > 0.05). Structural asymmetry of the calcaneus is established at an early stage to satisfy the mechanical requirements of a short rigid lever [18]. This may be due to the innate need of wild artiodactyls to avoid predators.

In addition, among mature *S. tatarica* (captive wild artiodactyl), *O. hemionus hemionus* (wild artiodactyl), and *O. aries* (domesticated artiodactyl), *S. tatarica* had the largest Imax/Imin (raw data: DS: 3.05, MS: 7.36) (Figure-S4) [4, 7, 9], data for *O. hemionus hemionus* and *O. aries* are derived from Kopp and Differences in Imax/Imin between mature *S. tatarica* and *O. hemionus hemionus* (DS: p = 0.468; MS: p = 0.275) were much lower than those between mature *S. tatarica* and *O. aries* (DS, and MS, p < 0.05). Mature *S. tatarica* and *O. hemionus hemionus* have greater cortical bone asymmetry than *O. aries*, which is consistent with bone strength adaptation under fast-running conditions. This characteristic of cortical bone distribution (i.e., in the calcaneus, Imax/Imin) is conserved in the short-term; thus, it may be genetically determined in the long-term.

**Figure-S4:**
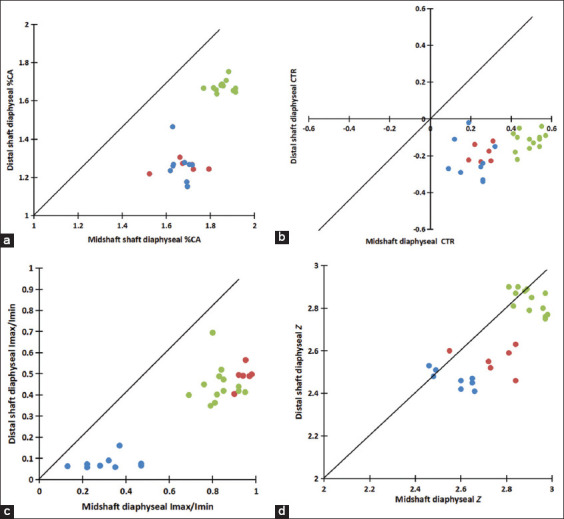
Comparison of the mid- and distal shafts (DS) of the calcaneus for each individual in the sample. The lines in each graph represent similarity between the mid- and DS calcaneal measurements for each variable. (a) Percent cortical bone area (%CA), (b) Cortical bone thickness ratio, (c) The ratio of principal moments of area (Imax/Imin), (d) Section modulus (Z). *Saiga tatarica* (red dot), *Odocoileus hemionus hemionus* (green dot), *Ovis aries* (blue dot). Data for *O. hemionus hemionus* and *O. aries* were derived from Kopp [4] and Skedros *et al*. [7, 9].

The previous studies have shown that mature *O. hemionus* *hemionus* have significantly greater area and thickness of the cortical bone at the MS of the calcaneus than mature *O. aries* [4, 7]. We found no significant differences in %CA and CTR at both diaphyseal locations of the calcaneus between mature *S. tatarica* and *O. aries* compared with a previous study [4, 6]. However, %CA at both diaphyseal locations and CTR in the MS of the calcaneus was significantly lower in mature *S. tatarica* than in mature *O. hemionus hemionus* (Figure-S4). We found significant differences in the %CA of *S. tatarica* and *O. aries* at both diaphyseal locations in immature individuals (Table-2); however, no significant differences were found in mature individuals. Similarly, the CTR at the MS of the calcaneus differed significantly between immature groups and then converged during adulthood. These results suggest that variation in the cross-sectional CA and cortical bone thickness may be critical phenotypes for adapting to habitual loading under different stress environments [19]. Skedros *et al*. [7] found similar growth rates in the cortical bone area during *O. hemionus hemionus* and *O. aries* development. However, in our study, cortical bone geometry growth rates during development were significantly different between *S. tatarica* and *O. aries*. The %CA growth rate was significantly higher in *S. tatarica* than in *O. aries* (MS: mature *S. tatarica* was 6.25% higher than immature *S. tatarica*; mature *O. aries* was 0.6% higher than immature *O. aries*, p < 0.05; DS; mature *S. tatarica* was 17.1% higher than immature *S. tatarica*; mature *O. aries* was 1.9% higher than immature *O. aries*, p < 0.05). This difference was also observed in the CTR at the MS of the calcaneus of both species (mature *S. tatarica* was 185.9% higher than immature *S. tatarica* and mature *O. aries* was 114.3 % higher than immature *O. aries*, p < 0.05). Consistent with Franklyn *et al*. [19] results, we found that these two indicators are sensitive to external environmental stress. The faster increase in %CA and CTR in *S. tatarica* calcanei might reflect its more rapid elevation of body mass, due to the abundant and rich captive diet in the zoo [20]. This is consistent with the law of limb bone development where body mass is the strongest single predictor of cross-sectional geometry [19]. These results suggest that environmental factors are more important than genetics in determining the %CA and CTR in the development of artiodactyls.

Franklyn *et al*. [19] suggested that *Z* is the key parameter in weight-bearing bone to reflect movement ability, which is thought to be highly correlated with %CA. However, the results of the present study show that *Z* is less sensitive to stress than expected, which might reflect the fact that *Z* is determined over evolutionary time (long-term adaptation).

To some extent, the %CA and CTR values indicate that captive wild *S. tatarica* possesses less movement ability than wild cursors. These results indicate that there was adaptive convergence in morphological variations of the calcaneus in artiodactyls with similar body designs and lifestyles. Nevertheless, we found a significant difference in CTR between immature individuals of the two species, but no significant difference between mature individuals of the two species, only in the MS, which might be due to the insertion of the Achilles tendon at the posterior (DS) of the calcaneus shielding the stress [4–6]. The extent of protection provided by these tissues and their effect on cortical bone thickness require further investigation.

### Variations in lifestyle-related trabecular regional morphology at the nanoscopic scale

Although cortical bone plays a major role in bearing mechanical loads with a limited amount of deformation, trabecular tracts that are continuous with these cortices can absorb and disperse the load transmitted from the adjacent cortical bone [13]. Therefore, the trabecular bone structure is critical for adapting to increased bone loading. The living environment affects the nanostructure and mechanical characteristics of the trabecular bone and directly reflects bone quality [21, 22]. In the present study, no significant differences in grain size or *E* were found between the two species at the level of the entire calcaneus in each age group (Table-3). However, there were significant differences in the localized regions of the calcaneus between the two species (Table-4). These results indicate that trabeculae morphology alterations between species occur on a considerably localized level. In such studies, it is necessary to consider all regions [4].

Analysis of the entire calcaneus and localized regions (dorsal and plantar regions of DS and MS) revealed that in both the overall and localized regions in *S. tatarica* calcaneus, grain size and *E* increased with age and RMS roughness decreased with age; the same trends were also found in *O. aries* calcaneus. Gao *et al*. [21] presented comparable results and suggested that the larger grain sizes observed in mature bones were caused by a delay in bone remodeling. Larger grain sizes have flatter surfaces and low surface roughness [23]. Regenerating bone has been found to possess a lower *E* value than mature bone [24]. These results suggest that the degree of bone mineralization increases with age and that bone strength increases as an individual matures from infancy to adulthood [23].

However, with the exception of *E* values in localized regions, measurements of the other two indicators did not support the assumption that there are significant differences between the two species in infancy, which converge during growth. Considering the similar tendencies of the mechanical parameters between the dorsal and plantar regions in the MS in both species, a ratio of *E* (*E* ratio = *E* of the plantar region/E of the dorsal region) was defined to evaluate the difference in the dorsal–plantar region (Table-5). The dorsal–plantar differences of *E* in the MS differed more between the immature groups (p < 0.05) than between the mature groups (p > 0.05), suggesting that the morphology of *S. tatarica* and *O. aries* converges in localized regions of the calcaneus during growth and development. Moreover, during ontogeny, the increased rate of *E* in the MS of the *S. tatarica* calcaneus was similar to that of %CA and CTR and was significantly greater than that of *O. aries* (dorsal region: Mature *S. tatarica* is 55.4% higher than immature *S. tatarica*, mature *O. aries* is 2.7% higher than immature *O. aries*; and plantar region: Mature *S. tatarica* is 20.5% higher than immature *S. tatarica*, mature *O. aries* is 8.2% higher than immature *O. aries*, p < 0.05), suggesting that the *E* of trabecular bone is also sensitive to changes in external stress (such as weight load). Similar trends for changes in CA, asymmetric cortical thickness, and trabecular mechanical properties (i.e., *E*) may also reflect potential synergism between the trabecular and nearby cortical bone [25].

**Table-5:** Dorsal-plantar differences in *E* (*E* ratio) for the mid-shaft of the calcaneus by species.

Species	Immature bones			Mature bones		
	
	Saiga antelope	Sheep	p-value	Saiga antelope	Sheep	p-value
*E* ratio	1.48 (0.12)	1.01 (0.14)	0.021*	1.15 (0.09)	1.06 (0.10)	0.421

Mean values (standard deviations).

* Statistically significant differences between species (p < 0.05)

The mechanical properties of the trabecular bone in the plantar region of the MS of the artiodactyl calcaneus are weaker than those in the dorsal region of the MS and the plantar region in the DS, and the trabecular bone can be reinforced by structural and material adjustments to regions with poor mechanical properties [26]. Avoiding the formation of relatively large mineral grains is an effective way to improve the mechanical properties of materials [27]. In our study, *E* values in the plantar regions in the MS of the calcanei in both mature and immature *S. tatarica* were significantly greater than those in the corresponding regions of the DSs (p < 0.05). This indicates that the plantar regions in MSs with comparatively deficient mechanical properties were strengthened through structural changes in the trabecular bone, which were reflected in the mechanical behavior (i.e. *E*) of the trabeculae [28]. However, grains in the plantar region of the DS in mature *S. tatarica* were significantly smaller than those in the corresponding region in the MS, suggesting that reinforcement may be achieved through changes in the micro-architectural structures (such as anisotropy) of trabecular bone rather than adjustments in nanostructure and materials [29, 30]. Detailed studies using micro-CT should analyze the bone microarchitecture of trabecular bone. Therefore, grain size and RMS roughness are more likely to be determined by aging and genes than by short-term adaptations.

In addition, our results showed that *E* values in the dorsal MS of the calcanei were lower than those in the plantar MS, regardless of age class, in both species. These results are consistent with a previous study showing that the mechanical properties of the plantar trabecular tract of the artiodactyl calcaneus were inferior to those of the dorsal trabecular tract [4]. However, this phenomenon was not observed in the DS of the calcaneus. This indicates that due to the attachment of soft tissue and the Achilles tendon on the DS of the calcaneus, forces in the radial direction on the DS of the calcaneus are more complex than those on the MS, and the force direction of the dorsal “compression” region and plantar “tension” region on the DS is not as clear as that on the MS. Therefore, only the MS of the calcaneus may truly reflect functional bone adaptation.

Because *S. tatarica* is an endangered species in China, the sample size recruited in the present study was limited, which may explain the large SDs (e.g., RMS roughness). In addition, this study primarily compared the morphological differences between captive wild and domesticated artiodactyls. Descriptions of the adaptation of wild artiodactyls in captivity could be enhanced if morphological data on calcaneal bone in wild artiodactyls were introduced for comparison. However, the nanomorphological and mechanical characteristics of calcaneal trabeculae in wild artiodactyls have not yet been reported. Nonetheless, the adaptive changes in calcaneus morphology in captive-bred wild *S. tatarica* need to be investigated for its conservation. Therefore, domesticated *O. aries*, which are similar in size to *S. tatarica* and belong to the same family as *S. tatarica*, were selected as controls in this study given the convergence of populations living in the same habitat. For the 1^st^ time, the present study provides an evaluation of the nanomorphological and mechanical parameters of the calcaneus of *S. tatarica*, filling the gap in the study on artiodactyl nano-osteomorphology in the international anatomical research field. This study provides an important reference for functional bone adaptations in wild artiodactyl in captivity.

## Conclusion

We present the first study to investigate the morphology and mechanical characteristics of *S. tatarica* calcaneus. These results indicate that the calcaneus of wild artiodactyls can undergo potentially transitional changes during short-term adaptation to captivity. We found that %CA, CTR, and *E* in the MS were significantly different between *S. tatarica* and *O. aries* at a young age but not between mature individuals, suggesting that the calcaneus morphology of *S. tatarica* converges with that of domesticated *O. aries* in captivity during growth and development. This suggests that the calcaneus of wild artiodactyls can undergo potentially transitional changes after decades of captive breeding. %CA, CTR, and *E* are highly sensitive to changes in stress and can be preliminarily identified as morphological signs of functional bone adaptation in artiodactyls. Artiodactyls undergo rapid plastic (non-heritable) changes during their life. The *Z* of the calcaneal diaphysis, size of mineral grains, and surface roughness of the trabeculae were determined over evolutionary time or aging.

## Data Availability

The datasets used in this study are available from the corresponding author on reasonable request.

## Authors’ Contributions

LJ: Conceptualization, methodology, validation, formal analysis, resources, data curation, writing—review and editing, supervision, project administration. JX: Validation, resources. JC: Resources, data curation. SH: Data curation. XQ: Software. FW: Conceptualization, methodology, validation, investigation, formal analysis, data curation, writing—original draft preparation, writing—review and editing, visualization. All authors have read, reviewed, and agreed to the published version of the manuscript.
